# Use of dental services among adults from a birth cohort in the South region of Brazil

**DOI:** 10.11606/s1518-8787.2023057004604

**Published:** 2023-07-31

**Authors:** Rafaela do Carmo Borges, Mariana Silveira Echeverria, Sarah Arangurem Karam, Bernardo Lessa Horta, Flávio Fernando Demarco

**Affiliations:** I Universidade Federal de Pelotas Faculdade de Medicina Programa de Pós-graduação em Epidemiologia Pelotas RS Brasil Universidade Federal de Pelotas. Faculdade de Medicina. Programa de Pós-graduação em Epidemiologia. Pelotas, RS, Brasil.; II Universidade Federal de Pelotas Faculdade de Odontologia Programa de Pós-graduação em Odontologia Pelotas RS Brasil Universidade Federal de Pelotas. Faculdade de Odontologia. Programa de Pós-graduação em Odontologia. Pelotas, RS, Brasil.

**Keywords:** Dental Care, Dental Health Services, Disparities in Health Care, Adults

## Abstract

**OBJECTIVE:**

Measure the prevalence of use of dental services in the previous year and associated factors among 31-year-old adults from a birth cohort of 1982.

**METHODS:**

This is a cross-sectional study that analyzed a birth cohort of 1982 from the city of Pelotas. In 1997, a systematic sample of 27% of the city’s census sectors was defined and all households in these sectors were visited, where 1,076 15-year-old adolescents were interviewed. For the oral health studies, 900 of these individuals were randomly selected and followed up at 24 and 31 years of age. The study used data collected from 523 individuals in 2013 (at 31 years old). The outcome was visit to the dentist (use of dental services) in the previous year. Demographic factors (sex), socioeconomic factors (income, education), and oral health factors (reason and type of service, self-perception of oral health, dental pain and caries experience - DMFT) were used as independent variables. Prevalence ratios were estimated using Poisson regression.

**RESULTS:**

The prevalence of use of dental services in the previous year was 55.3% (95%CI: 51.0–59.5%). In the adjusted analysis, the reason and type of service, self-perception of oral health, and DMFT were associated with the outcome. A stronger association was found with use of dental services in individuals who visited for prevention and used the private service, who were satisfied with their oral health, and who had more caries experiences.

**CONCLUSION:**

55.3% of the cohort sample used dental services in the previous year. Individuals who visited the dentist of private service for preventive reasons, who were very satisfied with their oral health, used these services in a higher proportion. In addition, a higher DMFT index also led to higher use of services.

## INTRODUCTION

Around 3.5 billion people in the world have oral diseases, with caries being the most prevalent. According to the Global Burden of Disease Study, 2.3 billion people have permanent teeth affected by caries^1,2^. A strong relation was observed between the oral conditions of the population and their socioeconomic situation^3^. Oral diseases are an indicator of inequalities and health problems in the population, as they affect marginalized people in society in a different way. Lifestyle and type of work are part of these determinants and include elements such as housing, working conditions, support and access to health^3^. In Brazil, in the last national oral health survey, adults aged from 35 to 44 years had caries experience, with an average of 16.75 affected teeth. Missing teeth accounted for around 44.7% of the index in this age group. Also, among adults and elderly people, missing teeth due to caries was the most prevalent problem^4^.

According to the American Dental Association^5^, to maintain good oral health, it is recommended to visit the dentist regularly, with the interval between visits determined by the dentist according to the patient’s needs and history. Visiting the dentist frequently is important to prevent toothache, periodontal diseases, mouth cancer, missing teeth, among other problems^5^. Several studies conducted around the world have reported some patterns in the association of use of dental services and sociodemographic and oral health variables. In Europe, in countries such as the United Kingdom, Finland, and Ireland, women with higher income and higher educational level are positively associated with use of dental services^6^. In the United States and Colombia, people who reported good oral health were also more associated with recent use of dental services^9,10^. On the other hand, use of dental services was lower in China when compared to other places in the world. In this country, only 20.1% of adults visited the dentist in the year prior to the study and the most prevalent reason was curative treatment^11^. Nigeria also presented lower prevalence rates, as only 26.4% of individuals reported using the service at least once before the study, 54.9% of which for curative treatment^12^. Finally, in Paraguay, 11% of people reported visiting the dentist once a year and 64% only seek dental care only when required; that is, prevention is a service used by a minority^13^.

In 1998, the National Household Sample Survey (PNAD) reported low use of dental services and divergence between higher and lower income groups regarding the use of such services^14^. However, according to the 2003 and 2008 PNAD, such use increased and issues in service access decreased in this two-year period. More than 89 million people saw a dentist in the 12 months prior to the study, which corresponded to 44.4%. The highest rates of use of dental services in the previous year were observed in the South and Southeast regions of Brazil (51.9% and 48.3%, respectively) and the lowest were in the North and Northeast regions (34.4% and 37.5%, respectively)^15,16^. However, unequal use persists in different groups of society, such as people of low income and educational level^17,18^.

This study aimed to assess the prevalence of visits to the dentist (use of dental services) in the last 12 months among adults from a cohort of live births.

## METHODS

### Cohort of Live Births of 1982 – Pelotas, RS

**Figure 1:**
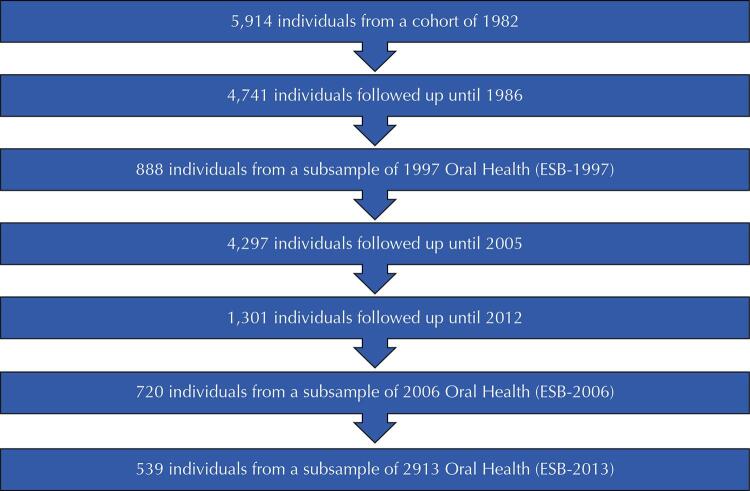
Flowchart with the main follow-ups of a cohort of live births in Pelotas, RS^21^.

In 1997, 1,079 participants were identified from the 1982 birth cohort by analyzing 70 census sectors out of a total of 259 in the city of Pelotas, RS, where the individuals were interviewed. For oral health studies, a random subsample of these 1,079 participants was selected with 900 individuals (ESB-97). The purpose of these studies was to identify the main oral diseases reported throughout life, assess the socioeconomic situation with oral health problems, and investigate the behaviors of individuals in relation to oral health. Follow-up was performed at 15 (n = 888), 24 (n = 720), and 31 years of age^19^. This sample was sufficient to estimate the prevalence rates of unknown outcomes with 50% prevalence, margin of error of 5 percentage points, and 95% confidence intervals (95%CI)^20^. Figure 1 shows a flowchart illustrating general and oral health follow-up performed with the 1982 cohort over the years^21^.

Individuals participating in the ESB-97 were contacted (n = 888) in 2013 for the follow-up at 31 years of age and, of these, 539 were interviewed and examined. In this follow-up, oral hygiene habits, use of dental services, toothache in the last six months, and some oral problems were investigated. The sample from this follow-up was used in our study, with a cross-section of these data. Six dentists received theoretical and practical training and were calibrated to perform the exams, with the level of agreement measured by the Kappa coefficient, with 0.65 as the lowest value in our study. In the calibration process, 20 individuals of similar age, but not belonging to the cohort, were examined. Follow-up examinations were performed at the homes of participants. For quality control, 15% of the telephone interviews were repeated^20,22^. More details about the method of oral health studies with the cohort can be found in previous publications^19^.

### Outcome

To guide this study, the following question was proposed: “When was your last visit to a dentist?” The response categories were: “one year or less,” “between one and two years,” and “more than two years.” It was adapted to: “Did you see the dentist in the last year? (12 months prior to the visit)”, with yes or no answers.

### Explanation

The outcome was described according to sociodemographic characteristics, such as the sex (male and female), skin color (white, black/brown, other), education (years of study), and income (in income quintiles).

Use of dental services was also described according to oral health characteristics: toothache in the last six months (yes or no), reason of visit (preventive or curative), and type of service (public or private/health plan); in addition to self-perception of oral health (very satisfied/satisfied or neither satisfied/nor dissatisfied/dissatisfied/very dissatisfied); and mean index of decayed, missing, or filled teeth (DMFT) collected in the clinical examination.

### Data Analysis

The theoretical pattern used in the analysis of variables was based on the model proposed by Andersen^34^. The socioeconomic and demographic variables are at the most distal level of this study, such as sex, income, education, and skin color. The factors related to the individual’s health beliefs are at the intermediate level, which define the actions in relation to health services, such as the type of service and the reason of the last visit. Finally, oral health conditions are considered at the most proximal level, which, in this study, were: pain in the last 6 months, self-perception of oral health, and caries experience.

**Figure 2:**
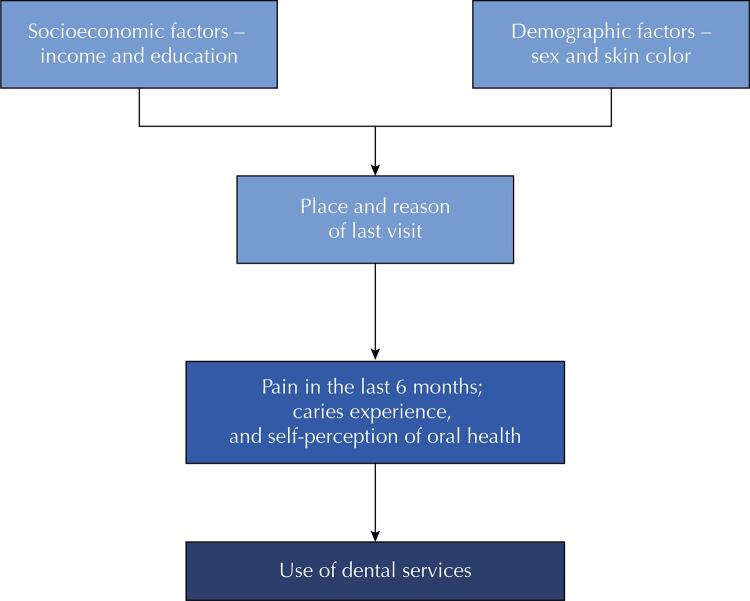
Data analysis model.

Data were analyzed using Stata 14.2 statistical software. A descriptive analysis was performed with absolute and relative frequencies of all variables, based on the model illustrated in Figure 2 described above. Bivariate and multivariate Poisson regressions with robust variance were performed to test the association between use of dental services in the previous year and other covariates, respecting the hierarchical model. Variables of the first level were inserted in the regression, variables with p > 0.2 were removed, and the level was processed again until all variables presented p > 0.2; then variables of the second level and then variables of the third level were inserted using the same logic. Prevalence ratio (PR) and respective 95% confidence intervals were estimated. The significance level considered was 5% for all statistical tests.

### Ethical Aspects

This study was approved by the Ethics Committee of the Faculdade de Medicina at the Universidade Federal de Pelotas (UFPel). All participants signed an informed consent form and had the guarantee of data confidentiality^23^.

## RESULTS

### Sample of Adults

Of all 523 individuals in the sample, most were white (78.6%), with an average income of BRL 3,118.77 (± 2,825.13), and mean education of 11.5 years (± 3.99). Among all participants, 55.3% had visited the dentist in the previous year. Regarding the type of service and reason of the last visit, most used the private service (77.1%) for curative reasons (66.4%).

Regarding pain, 31.2% felt it in the last six months. DMFT was 7.1, and most of them were dissatisfied or indifferent (54.6%) in relation to self-perception of oral health. Table 1 shows sample details.

**Table 1:** Description of the sample of 31-year-old adults from a 1982 cohort of live births in the city of Pelotas, RS, according to demographic, socioeconomic, and oral health characteristics. Pelotas, Rio Grande do Sul, Brazil, 2020.

Variables (n)	n	%
Sex (523)		
Female	262	50.1
Male	261	49.9
Skin color (495)		
White	389	78.6
Black/brown	92	18.6
Other	14	2.8
Income (456)		
Q1	70	15.4
Q2	97	21.3
Q3	101	22.1
Q4	105	23
Q5	83	18.2
Education (478)	11.5^a^	4.0^b^
Dental visit in the previous year (523)		
No	234	44.7
Yes	289	55.3
Reason of dental visit (446)		
Preventive	150	33.6
Curative	296	66.4
Type of service in the last visit (525)		
Public	120	22.9
Private/health plan	405	77.1
Pain in the last 6 months (520)		
No	358	68.8
Yes	162	31.2
Self-perception of oral health (537)		
Satisfied/very satisfied	244	45.4
Neither satisfied/nor dissatisfied/dissatisfied/very dissatisfied	293	54.6
DMFT (539)	7^a^	4.5^b^

DMFT: decayed, missing and filled teeth.

^a^ Mean value.

^b^ Standard deviation.

### Use of Dental Services in the Previous Year

In the crude analysis, the factors that were positively associated with use of dental services in the previous year were sex (female individuals), higher income, and higher levels of education. Regarding oral health, the factors with a significant association were: seeing a dentist for preventive reasons, using the private service, and having a good self-perception of oral health. Finally, those with a higher DMFT index showed a stronger association with use of dental services in the previous year.

In the adjusted analysis for possible confounders, the following factors were associated with the outcome: reason and type of service in the last visit, self-perception of oral health, and DMFT. The prevalence of visits in the previous year was 79% higher among individuals who used the private service when compared to those who used the public service, and 26% higher among those who visited a dentist for preventive reasons when compared to those who visited for curative reasons. Individuals who perceived their oral health satisfactorily had a 27% higher prevalence of visits than those who were indifferent or dissatisfied regarding their oral health. Finally, people with more caries experiences had a higher prevalence of visits to the dentist in the previous year, with prevalence increasing 3% for every decayed, missing, or filled decayed tooth. Table 2 shows the crude and adjusted analyses.

**Table 2:** Prevalence ratios (PR) and 95% confidence intervals (95%CI) of crude and adjusted analyses of use of dental services in the previous year according to demographic, socioeconomic, and oral health variables of 31-year-old adults from a 1982 birth cohort. Pelotas, Rio Grande do Sul, Brazil, 2020.

Variables	Use of dental services in the previous year			

	Crude analysis		Adjusted analysis^a^	
	
	PR (95%CI)	p-value	PR (95%CI)	p-value
Sex (523)		0.003		0,084
Male	1		1	
Female	1.27 (1.09–1.49)		1.17 (0.98–1.39)	
Skin color (495)		0.252		-
White	1		-	
Black/brown	0.82 (0.65–1.03)		-	
Other	0.98 (0.62–1.56)		-	
Income (456)		0.007		
Q1	1		-	-
Q2	1.23 (0.88–1.70)		-	
Q3	1.22 (0.88–1.70)		-	
Q4	1.44 (1.06–1.97)		-	
Q5	1.63 (1.20–2.21)		-	
Education (478)	1.04 (1.02–1.07)	< 0.001	1.01 (0.99–1.04)	0.344
Reason of dental visit (445)		< 0.001		0.003
Preventive	1		1	
Curative	0.64 (0.54–0.76)		0.74 (0.61–0.90)	
Type of service in the last visit (521)		< 0.001		< 0.001
Public	1		1	
Private/health plan	2.10 (1.58–2.80)		1.79 (1.31–2.45)	
Pain in the last 6 months (507)		0.052		0.55
No	1		1	
Yes	0.84 (0.70–1.00)		1.07 (0.86 – 1.33)	
Self-perception of oral health (523)		< 0.001		0.001
Satisfied/very satisfied	1		1	
Neither satisfied/nor dissatisfied/dissatisfied/very dissatisfied	0.70 (0.60–0.81)		0.73 (0.60–0.89)	
DMFT (539)	1.02 (1.01–1.04)	0.006	1.03 (1.01–1.05)	0.006

95%CI: 95% confidence inteval; DMFT: decayed, missing and filled teeth.

^a^ Analysis adjusted for sex, skin color, education, income, reason, and place of last visit, self-perception of need and reason, pain in the last 6 months, self-perception of oral health, and DMFT.

## DISCUSSION

The prevalence of use of dental services in the previous year in this study was 55.3% (95%CI: 51.0–59.5%), a little higher than the rate reported in the literature for the age group studied. In the 2010 National Oral Health Survey, this number was 49.1% for the country in general and in the 2013 National Health Survey (PNS), 44.4%^4^. In the same report, Rio Grande do Sul had a proportion of 52.7%, higher than the national rate. In Pelotas, a population-based study was conducted in 2006, in which the prevalence of use of dental services in the previous year was 50.9%, but this study also included adolescents and elderly individuals, in addition to adults^24^. Data from our study confirm the epidemiological profile described in other studies with adults from the region where Pelotas is located^7,10,24^.

White women, with higher income and education level, are more associated with the use of dental services in the literature, but these associations were not significant in our study. Herkrath et al.^17^ used data from the 2013 PNS and presented similar results to those in the literature: people with higher education were associated with more recent use of services than those with lower educational level. In general, women are associated with more frequent use of dental services than men^16,24,25^. The occupation level shows higher proportions of men than women, which may explain this characteristic^27^, as men find more trouble visiting the dentist during working hours^28,29^. The potential lack of association between education and use of dental services could be linked with two factors: 1) average education of 11.5 years, i.e., it is a relatively educated sample; 2) the sample size may have been insufficient to detect potential associations, which is also a factor for the lack of association for sex, skin color, and income.

Regarding the type and reason for using the dental service, private service was the most common type and preventive service was the most frequent reason. The prevalence of use was 80% higher in the private service when compared to public services. In a study about the use of medical and dental services in the previous year, which used PNAD data from 1998, 2003, and 2008, as well as data from the 2013 PNS, a trend was observed towards an increase in services over the years among individuals with or without a private health plan, but individuals with a health plan had higher percentage of use of services in all years, that is, adults with a health plan had a higher chance of using services in all years analyzed^30^. Income influences the type of service, and people who use public services find it more difficult to pay for other services. Barros and Bertoldi^14^ highlight that the adult population is historically neglected, without priority in general health care in public services.

Satisfaction in self-perception of oral health is often associated with a higher frequency of visits to the dentist. Among the 31-year-old individuals in this study, use of dental services was 26% higher among those who were satisfied with their own oral health when compared to those feeling indifferent or dissatisfied with it. Self-perception is a factor based on health belief, which influences the use of dental services. Individual who considers their oral health as good or very good are the same individuals associated with more frequent use^17,24^.

The DMFT index was also associated with more frequent use, with the prevalence of use increasing 3% for every decayed, missing or filled tooth. Missing tooth was one of the factors associated with the use of services in the study by Herkrath et al.^17^ in 2018. Few studies have been conducted addressing the association between use and the DMFT index in adults. Most studies on this topic involve populations of children and adolescents. Provision of health services, together with the perception of treatment need, can define the use of dental services^33^. Therefore, just visiting the dentist may not result in a decrease in oral problems^24^. Possibly people with higher DMFT index went to the dentist because they needed a treatment, i.e., because they had more caries experience, although in our study, recent use was more associated with preventive than curative reasons. The most immediate cause for using health services is the individual’s perception of his or her own need for treatment^31,34^ and, in the general population, this perception is related to the symptoms and social and functional problems resulting from oral diseases^24^.

One of the strong points of this study was the association of clinical examination with sociodemographic variables and use of dental services in the previous year in adults. When studying this population, questionnaires are usually developed about factors related to the use of services, but they often do not cover the clinical examination. Study limitations include its design, because despite being a cohort study, it is a cross-sectional analysis. Also, the outcome was analyzed through self-report, which may overestimate data, considering that visiting a dentist is a socially acceptable behavior.

Another aspect is that, although the Andersen’s model was used, not all variables were used because some had not been collected. This study analyzed a subsample from a cohort of 1982, assessing the oral health of 888 individuals aged 15 years. In the follow-up at 31 years (2013), 523 individuals were evaluated regarding the outcome, which corresponded to almost 60% of the initial subsample. This reduced sample size may have been the cause of absent association with some variables, due to lower power. However, the sample analyzed at age 31 has similar characteristics to the sample evaluated at age 15, considering demographic and socioeconomic factors, and that the subsample of oral health has characteristics of the entire cohort (data not shown).

People who visited a dentist for curative reasons, in the public service, who were indifferent or dissatisfied with their oral health, and had fewer caries experiences were the ones who used dental services with the lowest frequency. People who use public services and are dissatisfied with their oral health need new social actions to support existing policies and include disadvantaged groups. Our study shows public dental services should be expanded to enable access to dental treatment for vulnerable groups with lower oral health indicators. Considering the impact of social inequality on oral health, strategies to expand access for disadvantaged groups are critical for improving oral health indicators.
