# Supplementary material for: Use of dental services among adults from a birth cohort in the South region of Brazil
Source: Rev Saude Publica. 2023 Jul 31;57:47. doi: 10.11606/s1518-8787.2023057004604 (PMC10392773; doi:10.11606/s1518-8787.2023057004604)
Supplement: Translation

# Translation: Uso de serviços odontológicos em adultos de uma coorte de nascimentos no sul do Brasil

**Authors:** Rafaela do Carmo Borges, Mariana Silveira Echeverria, Sarah Arangurem Karam, Bernardo Lessa Horta, Flávio Fernando Demarco

**Affiliations:** I Universidade Federal de Pelotas Faculdade de Medicina Programa de Pós-graduação em Epidemiologia Pelotas RS Brasil Universidade Federal de Pelotas. Faculdade de Medicina. Programa de Pós-graduação em Epidemiologia. Pelotas, RS, Brasil.; II Universidade Federal de Pelotas Faculdade de Odontologia Programa de Pós-graduação em Odontologia Pelotas RS Brasil Universidade Federal de Pelotas. Faculdade de Odontologia. Programa de Pós-graduação em Odontologia. Pelotas, RS, Brasil.

**Keywords:** Assistência Odontológica, Serviços de Saúde Bucal, Disparidades em Assistência à Saúde, Adultos

## Abstract

**OBJETIVO:**

Mensurar a prevalência de uso de serviços odontológicos no último ano e os fatores associados em adultos de 31 anos pertencentes à coorte de nascimentos de 1982.

**MÉTODOS:**

Estudo transversal, aninhado na coorte de nascimentos de Pelotas de 1982. Em 1997, uma amostra sistemática de 27% dos setores censitários da cidade foi realizada e todos os domicílios desses setores foram visitados, onde 1.076 adolescentes de 15 anos foram entrevistados. Para os estudos de saúde bucal foram sorteados aleatoriamente 900 desses indivíduos, que foram acompanhados também aos 24 e 31 anos. O estudo utilizou dados coletados de 523 indivíduos em 2013 (aos 31 anos). O desfecho foi a ida ao dentista (uso do serviço) no último ano. Fatores demográficos, (sexo), socioeconômicos (renda, escolaridade) e de saúde bucal (motivo e tipo de serviço da consulta, autopercepção de saúde bucal, dor e experiência de cárie - CPOD) foram utilizados como variáveis independentes. As razões de prevalência foram estimadas usando a regressão de Poisson.

**RESULTADOS:**

A prevalência de uso de serviços odontológicos no último ano foi de 55,3% (IC95%: 51,0 –59,5%). Na análise ajustada, o motivo e tipo de serviço da consulta, a autopercepção de saúde bucal e o CPOD foram associados ao desfecho. Foi encontrada maior associação com a utilização de serviços odontológicos em indivíduos que visitaram por prevenção e usaram o serviço privado, satisfeitos com a sua saúde bucal e que tinham maior experiência de cárie.

**CONCLUSÃO:**

55,3% da amostra da coorte utilizaram os seviços odontológicos no último ano. Os indivíduos que visitaram o dentista por motivo preventivo, em consulta privada, que estavam muito satisfeitos ou satisfeitos com sua saúde bucal utilizaram em maior proporção esses serviços. Além disso, o maior índice de CPOD também levou ao maior uso.

## INTRODUÇÃO

Aproximadamente 3,5 bilhões de pessoas no mundo sofrem com doenças bucais, sendo a cárie a mais prevalente. De acordo com o estudo global de doenças, 2,3 bilhões de pessoas têm dentes permanentes afetados por cárie^1,2^. Existe uma forte relação entre as condições orais da população e sua situação socioeconômica^3^. As doenças bucais servem, de certa forma, como um indicador de desigualdades e problemas de saúde na população, visto que afetam diferentemente as pessoas marginalizadas na sociedade. O modo de viver e trabalhar faz parte desses determinantes e inclui elementos como moradia, condições de trabalho, suporte e acesso à saúde^3^. No Brasil, no último levantamento nacional de saúde bucal, adultos entre 35 e 44 anos apresentaram experiência de cárie, com média de dentes afetados de 16,75. Dentes perdidos foram responsáveis por aproximadamente 44,7% do índice nessa faixa de idade. Além disso, entre adultos e idosos, perda dentária por cárie foi o problema mais prevalente^4^.

Segundo a *American Dental Association*^5^, para a manutenção de uma boa saúde bucal, a recomendação é consultar o dentista de forma regular, sendo o intervalo entre as consultas determinado pelo profissional de acordo com a necessidade e o histórico de cada paciente. Ir ao dentista frequentemente é fundamental para prevenir dor de dente, doenças periodontais, câncer de boca, perda dentária, entre outros agravos^5^. Diversos estudos feitos ao redor do mundo demonstraram alguns padrões entre a associação da utilização de serviços odontológicos e variáveis sociodemográficas e de saúde bucal. Na Europa, em países como Reino Unido, Finlândia e Irlanda, mulheres com maior renda e maior escolaridade estão positivamente associadas ao uso do serviço^6^. Nos Estados Unidos e na Colômbia, pessoas que relataram ter uma boa saúde bucal também foram mais associadas ao uso recente do serviço odontológico^9,10^. Por outro lado, houve menor utilização em comparação com outros locais no mundo na China, onde apenas 20,1% dos adultos pesquisados foram ao dentista no ano anterior ao estudo e o motivo mais prevalente foi o curativo^11^. A Nigéria também apresentou prevalências mais baixas, pois apenas 26,4% dos indivíduos relataram ter feito uso do serviço pelo menos uma vez antes da pesquisa, dos quais 54,9% foram realizar procedimentos curativos^12^. Por fim, no Paraguai, 11% das pessoas relataram ir ao dentista uma vez ao ano, e 64% só busca atendimento odontológico se sentir necessidade, ou seja, prevenção é minoria^13^.

Em 1998, a Pesquisa Nacional por Amostra de Domicílios (PNAD) relatou baixa utilização de serviços odontológicos e divergência entre grupos de maior e menor renda quanto a esse uso^14^. Já nos dados da PNAD de 2003 e 2008, a utilização aumentou e a falta de acesso diminuiu entre esses dois anos de comparação. Mais de 89 milhões de pessoas se consultaram nos 12 meses anteriores à pesquisa, o que correspondia a 44,4%. As maiores proporções da utilização de serviço odontológico no último ano foram registradas nas regiões Sul e Sudeste (51,9% e 48,3%, respectivamente) e as menores foram nas regiões Norte e Nordeste (34,4% e 37,5%, respectivamente)^15,16^. Porém, ainda persistem desigualdades na utilização em grupos diversos da sociedade, como em pessoas de baixa renda e escolaridade^17,18^.

O objetivo desse trabalho é verificar a prevalência de visita ao dentista (uso dos serviços odontológicos) nos últimos 12 meses em adultos pertencentes a uma coorte de nascidos vivos.

## MÉTODOS

### Coorte de Nascidos Vivos de 1982 – Pelotas/RS

Em 1997, foram identificados 1.079 dos participantes da coorte de nascimentos de 1982, através de censo com 70 setores censitários de um total de 259 existentes na cidade de Pelotas/RS, onde os indivíduos foram entrevistados. Para os estudos de saúde bucal, foi feita uma subamostra aleatória desses 1.079 indivíduos, com 900 selecionados (ESB-97). A finalidade desses estudos foi pesquisar as principais doenças bucais que acontecem ao longo da vida e avaliar a situação socioeconômica com agravos de saúde bucal, além de investigar os comportamentos dos indivíduos em relação a esta. Os acompanhamentos foram realizados aos 15 anos (n = 888) e aos 24 anos (n = 720) e aos 31^19^. Essa amostra foi suficiente para estimar as taxas de prevalência dos desfechos desconhecidos com prevalência de 50%, com margem de erro de cinco pontos percentuais e intervalos de confiança de 95% (IC95%)^20^. A Figura 1 apresenta um fluxograma com acompanhamentos gerais e de saúde bucal realizados na coorte de 1982 ao longo dos anos^21^.

**Figura 1:** Fluxograma com os principais acompanhamentos da coorte de nascidos vivos de Pelotas-RS21.

Para o acompanhamento aos 31 anos, em 2013, os indivíduos participantes do ESB-97 foram contatados (n = 888) e, destes, 539 foram entrevistados e examinados. Nesse acompanhamento investigaram-se hábitos de higiene bucal, utilização de serviços odontológicos, dor de dente nos últimos seis meses e alguns problemas bucais. A amostra desse acompanhamento foi utilizada neste estudo, sendo um corte transversal desses dados. Seis dentistas receberam treinamento teórico e prático, e foram calibrados para a realização dos exames, com nível de concordância medido pelo coeficiente Kappa, sendo 0,65 o menor valor neste estudo. Na calibração foram examinados 20 indivíduos com idade similar, porém não pertencentes à coorte. Os exames do acompanhamento foram realizados nas residências dos participantes. Para o controle de qualidade, repetiram-se 15% das entrevistas por telefone^20,22^. Mais detalhes sobre a metodologia dos estudos de saúde bucal da coorte podem ser encontrados em publicações prévias^19^.

### Desfecho

Para orientar esta pesquisa, propôs-se a seguinte questão: “Quando você consultou o dentista pela última vez?”. As categorias de resposta eram: “um ano ou menos”; “entre um e dois anos”; e “mais de dois anos”. Isso foi dicotomizado em “Foi ao dentista no último ano? (12 meses anteriores a consulta)”, com respostas sim ou não.

### Exposições

O desfecho foi descrito segundo características sociodemográficas, como sexo do indivíduo (masculino e feminino), cor da pele (branca, preta/parda ou outras), escolaridade (em anos de estudo) e renda (em quintis de renda).

A utilização também foi descrita segundo as características de saúde bucal: dor de dente nos últimos seis meses (sim ou não), motivo da consulta (preventivo ou curativo) e tipo de serviço da consulta (público ou privado/convênio), além da autopercepção de saúde bucal (muito satisfeito/satisfeito ou nem satisfeito/nem insatisfeito/insatisfeito/muito insatisfeito) e a média do índice de dentes cariados, perdidos ou obturados (CPOD) coletados no exame clínico.

### Análise de dados

O padrão teórico utilizado para análise das variáveis foi baseado no modelo proposto por Andersen^34^. No nível mais distal desse estudo estão as variáveis socioeconômicas e demográficas, como sexo, renda, escolaridade e cor da pele. No nível intermediário estão inseridos fatores relacionados às crenças em saúde do indivíduo, que definem como serão as ações em relação aos serviços de saúde, como o tipo de serviço e o motivo da última consulta. Por fim, no nível mais proximal, são consideradas as condições de saúde bucal, que, neste estudo, foram: dor nos últimos 6 meses, autopercepção de saúde bucal e experiência de cárie.

Os dados foram analisados através do programa estatístico Stata 14.2. Uma análise descritiva foi feita com as frequências absolutas e relativas de todas as variáveis, com base no modelo demonstrado na Figura 2, descrito anteriormente. Regressões de Poisson bivariadas e multivariadas com variância robusta foram realizadas para testar a associação entre a utilização de serviços odontológicos no último ano e as demais covariáveis, respeitando o modelo hierárquico. As variáveis pertencentes ao primeiro nível foram inseridas ao mesmo tempo na regressão, aquelas variáveis com p > 0,2 foram retiradas e o nível foi rodado novamente até todas as variáveis possuírem valor de p < 0,2; logo após foram inseridas as do segundo e depois as do terceiro nível, seguindo a mesma lógica. Foram estimadas medidas de razão de prevalência (RP) e respectivos intervalos de confiança de 95%. O nível de significância considerado foi de 5% para todos os testes estatísticos.

**Figura 2:** Modelo da análise de dados.

### Aspectos Éticos

Esse estudo foi aprovado pelo Comitê de Ética da Faculdade de Medicina da Universidade Federal de Pelotas (UFPel) e todos os participantes assinaram termo de consentimento livre e esclarecido, com garantia de sigilo de suas informações^23^.

## RESULTADOS

### Amostra de Adultos

Dos 523 indivíduos da amostra, a maioria era de cor branca (78,6%), com média de renda de 3.118,77 reais (± 2.825,13) e média de 11,5 anos (± 3,99) de estudo.

Dos participantes da pesquisa, 55,3% haviam ido ao dentista no último ano. Quanto ao tipo de serviço e motivo da última consulta, a maioria utilizou o serviço privado (77,1%) e procurou o serviço por motivos curativos (66,4%).

Quanto à dor, 31,2% a sentiram nos últimos seis meses. A CPOD foi de 7,1, e a maioria demostrou-se insatisfeita ou indiferente (54,6%) em relação à autopercepção de saúde bucal. Mais detalhes da descrição da amostra podem ser conferidos na Tabela 1.

**Tabela 1:** Descrição da amostra de adultos com 31 anos de idade pertencentes à coorte de nascidos vivos de 1982 do município de Pelotas/RS segundo características demográficas, socioeconômicas e de saúde bucal. Pelotas, Rio Grande do Sul, Brasil, 2020.

Variáveis (n)	n	%
Sexo (523)		
Feminino	262	50,1
Masculino	261	49,9
Cor da pele (495)		
Branca	389	78,6
Preta/parda	92	18,6
Outros	14	2,8
Renda (456)		
Q1	70	15,4
Q2	97	21,3
Q3	101	22,1
Q4	105	23
Q5	83	18,2
Escolaridade (478)	11,5^a^	4,0^b^
Consulta odontológica no último ano (523)		
Não	234	44,7
Sim	289	55,3
Motivo da última consulta (446)		
Preventivo	150	33,6
Curativo	296	66,4
Tipo de serviço da última consulta (525)		
Público	120	22,9
Privado/convênio	405	77,1
Dor nos últimos 6 meses (520)		
Não	358	68,8
Sim	162	31,2
Autopercepção de saúde bucal (537)		
Muito satisfeito/satisfeito	244	45,4
Nem satisfeito/nem insatisfeito/insatisfeito/muito insatisfeito	293	54,6
CPOD (539)	7^a^	4,5^b^

CPOD: dentes cariados, perdidos ou obturados.

^a^ Média

^b^ Desvio padrão

### Utilização de Serviços Odontológicos no Último Ano

Na análise bruta, os fatores que apresentaram associação positiva com o uso de serviços odontológicos no último ano foram ser do sexo feminino, ter renda mais alta e níveis de escolaridade mais elevados. Quanto à saúde bucal, os fatores com associação significativa foram: ir à consulta com motivo preventivo, utilizar o serviço privado e apresentar boa autopercepção de saúde bucal. E, finalmente, quem possuía mais dentes cariados, perdidos ou obturados apresentou maior associação com a utilização no último ano.

Na análise ajustada para possíveis confundidores, continuaram associadas ao desfecho: motivo e tipo de serviço da última consulta, autopercepção de saúde bucal e CPOD. A prevalência de consulta no último ano foi 79% maior entre as pessoas que consultaram o serviço privado em comparação às que buscaram o serviço público, e 26% maior entre aquelas que o fizeram por motivos preventivos em comparação com aqueles que se consultaram por motivos curativos. Os indivíduos que percebiam sua saúde bucal de forma satisfatória tiveram prevalência de consulta 27% maior do que aqueles que avaliaram sua saúde bucal de forma indiferente ou insatisfatória. Por fim, pessoas com maior experiência de cárie apresentaram maiores prevalências de ida ao dentista no último ano, de modo que a prevalência aumenta 3% a cada aumento de 1 dente cariado perdido ou obturado. As análises bruta e ajustada podem ser conferidas na Tabela 2.

**Tabela 2:** Razões de prevalência (RP) e intervalos de confiança de 95% (IC95%) da análise bruta e ajustada da utilização de serviços odontológicos no último ano segundo variáveis demográficas, socioeconômicas e de saúde bucal dos indivíduos adultos de 31 anos da coorte de nascimentos de 1982. Pelotas, Rio Grande do Sul, Brasil, 2020.

Variáveis	Utilização de serviços odontológicos no último ano			
	Análise bruta		Análise ajustada^a^	
	RP (IC95%)	p-valor	RP (IC95%)	p-valor
Sexo (523)		0,003		0,084
Masculino	1		1	
Feminino	1,27 (1,09–1,49)		1,17 (0,98–1,39)	
Cor da pele (495)		0,252		-
Branca	1		-	
Preta/parda	0,82 (0,65–1,03)		-	
Outros	0,98 (0,62–1,56)		-	
Renda (456)		0,007		
Q1	1		-	-
Q2	1,23 (0,88–1,70)		-	
Q3	1,22 (0,88–1,70)		-	
Q4	1,44 (1,06–1,97)		-	
Q5	1,63 (1,20–2,21)		-	
Escolaridade (478)	1,04 (1,02–1,07)	< 0,001	1,01 (0,99–1,04)	0,344
Motivo da última consulta (445)		< 0,001		0,003
Preventivo	1		1	
Curativo	0,64 (0,54–0,76)		0,74 (0,61–0,90)	
Tipo de serviço da última consulta (521)		< 0,001		< 0,001
Público	1		1	
Privado/convênio	2,10 (1,58–2,80)		1,79 (1,31–2,45)	
Dor nos últimos 6 meses (507)		0,052		0,55
Não	1		1	
Sim	0,84 (0,70–1,00)		1,07 (0,86 – 1,33)	
Autopercepção de saúde bucal (523)		< 0,001		0,001
Muito satisfeito/satisfeito	1		1	
Nem satisfeito/nem insatisfeito/insatisfeito/muito insatisfeito	0,70 (0,60–0,81)		0,73 (0,60–0,89)	
CPOD (539)	1,02 (1,01–1,04)	0,006	1,03 (1,01–1,05)	0,006

IC95%: intervalo de confiança de 95%; CPOD: dentes cariados, perdidos ou obturados.

^a^ Análise ajustada para sexo, cor da pele, escolaridade, renda, motivo e local da última consulta, necessidade e motivo autopercebidos, dor nos últimos 6 meses, autopercepção de saúde bucal e CPOD.

## DISCUSSÃO

A prevalência de utilização de serviços odontológicos no último ano nesse estudo foi de 55,3% (IC95%: 51,0–59,5%), um número discretamente maior do que o relatado na literatura, geralmente, para a faixa etária estudada. Na Pesquisa Nacional de Saúde Bucal de 2010 esse número foi de 49,1% para o país em geral e em 2013; na Pesquisa Nacional de Saúde (PNS), foi 44,4%^4^. Na mesma pesquisa, o Rio Grande do Sul apresentou uma proporção de 52,7%, mais alta que a nacional. Em Pelotas foi realizado um estudo, em 2006, de base populacional, no qual a prevalência de utilização no último ano foi de 50,9%, porém esse estudo também englobou adolescentes e idosos, além dos adultos^24^. Os dados observados em nosso estudo confirmam o perfil epidemiológico descrito em outras pesquisas com a população adulta na região em que está inserida a cidade de Pelotas^7,10,24^.

Mulheres brancas, com maior renda e nível de escolaridade estão mais associadas à utilização de serviços odontológicos na literatura, porém essas associações não foram significativas nesse estudo. Herkrath et al.^17^ utilizaram os dados da PNS em 2013 e apresentaram resultados semelhantes aos da literatura em geral: pessoas com maior escolaridade foram associadas à utilização mais recente dos serviços do que as com menor escolaridade. As mulheres geralmente são associadas ao maior uso de serviço odontológico do que os homens^16,24,25^. O nível de ocupação apresenta maiores proporções de homens do que mulheres, o que pode explicar essa característica^27^. É possível que haja maior dificuldade por parte do sexo masculino em conseguir atendimento em serviços de saúde pelo horário de trabalho^28,29^. A potencial falta de associação entre escolaridade e uso de serviços poderia estar vinculada a dois fatores: 1) média de escolaridade de 11,5 anos, ou seja, é uma amostra relativamente escolarizada; 2) o tamanho amostral poder ter sido insuficiente para detectar potenciais associações, sendo esse também um fator para a falta de associação para sexo, cor da pele e renda.

Em relação ao tipo e motivo de uso do serviço odontológico, a maior utilização foi no serviço privado, com motivo preventivo de consulta. A prevalência de uso foi 80% maior no serviço privado que no serviço público. Em estudo sobre uso de serviços médicos e odontológicos no último ano, que utilizou dados da PNAD de 1998, 2003 e 2008, assim como dados da PNS de 2013, foi observada tendência de aumento dos serviços ao longo dos anos com ou sem plano privado, mas pessoas que tinham plano de saúde tiveram o maior percentual de uso dos serviços em todos os anos, ou seja, adultos com plano tiveram chance maior de utilização em todos os anos pesquisados^30^. A renda tem influência sobre o tipo de serviço utilizado, e pessoas que utilizam serviços públicos tem maior dificuldade de pagar outros serviços. Barros e Bertoldi^14^ apontam que a população adulta é historicamente negligenciada quanto à prioridade de atenção na saúde geral nos serviços públicos.

A satisfação na autopercepção de saúde bucal é geralmente associada a maior frequência de idas ao dentista. Nos indivíduos de 31 anos desse estudo, a utilização foi 26% maior entre aqueles que estavam satisfeitos com a sua própria saúde bucal quando comparados àqueles indiferentes ou insatisfeitos com ela. A autopercepção é um fator baseado na crença em saúde, e ela influencia no uso de serviço. Aquelas pessoas que apontam sua saúde bucal como boa ou muito boa são os mesmos associados à maior utilização^17,24^.

O índice de CPOD também foi associado ao maior uso, e a prevalência aumentou 3% na utilização a cada dente a mais cariado, perdido ou obturado. A perda dentária foi um dos fatores associados à utilização de serviços no estudo de Herkrath et al.^17^ em 2018. Há poucos estudos que abordam a associação entre o uso e o índice CPOD em adultos, a maior parte dos estudos sobre essa temática envolve populações de crianças e adolescentes. A oferta de serviços de saúde, juntamente com a percepção de necessidade de tratamento podem definir a utilização de serviços odontológicos^33^. E por isso, apenas a consulta ao dentista pode não resultar em diminuição dos agravos bucais^24^. Possivelmente as pessoas com maior índice de CPOD foram ao dentista por necessidade de realizar tratamento, ou seja, por possuírem mais experiência de cárie, ainda que no presente estudo a utilização recente tenha sido mais associada a indivíduos que consultaram por motivos preventivos do que curativos. A causa mais imediata para utilização dos serviços de saúde é a percepção do indivíduo da própria necessidade de tratamento^31,34^, e essa percepção está relacionada na população em geral com os sintomas e problemas sociais e funcionais gerados em decorrência de doenças bucais^24^.

Um dos pontos fortes deste estudo foi associar o exame clínico a variáveis sociodemográficas e à utilização de serviços odontológicos no último ano em adultos. Geralmente, quando se estuda essa população, são feitos questionários sobre fatores relacionados ao uso, porém estes geralmente não abrangem o exame clínico. Dentre as limitações da pesquisa, poderíamos considerar o seu desenho, pois, apesar de se tratar de estudo de coorte, a análise é transversal. Ainda, o desfecho foi realizado através do autorrelato, o que pode superestimar os dados, considerando que ir ao dentista é considerado um comportamento socialmente aceitável.

Outro ponto a destacar é que, apesar de o modelo de Andersen ter sido empregado, nem todas as variáveis foram utilizadas, pois algumas não tinham sido coletadas. O estudo foi realizado numa subamostra da coorte de 1982, com 888 indivíduos avaliados quanto à saúde bucal aos 15 anos. No acompanhamento realizado aos 31 anos (2013), 523 indivíduos foram avaliados quanto ao desfecho, o que corresponde a quase 60% da subamostra inicial. Esse tamanho amostral reduzido pode ser responsável pela falta de associação com algumas variáveis, em decorrência do menor poder. No entanto, é importante destacar que a amostra analisada aos 31 anos tem características similares à amostra avaliada aos 15 anos, levando-se em conta fatores demográficos e socioeconômicos, e que a subamostra de saúde bucal tem características da totalidade da coorte (dados não mostrados).

Pessoas que se consultaram por motivo curativo, no serviço público, indiferentes ou insatisfeitas com sua saúde bucal e com menor experiência de cárie, foram as que menos utilizaram os serviços odontológicos. Pessoas que frequentam o serviço público e estão insatisfeitos com a sua saúde bucal precisam de novas ações sociais, reforçando políticas já existentes, com inclusão dos grupos menos favorecidos. Nossos dados demonstram a necessidade de ampliação dos serviços odontológicos públicos, visando possibilitar o acesso dos indivíduos em maior vulnerabilidade e com piores indicadores de saúde bucal ao tratamento odontológico. Considerando o impacto que a desigualdade social tem sobre a saúde bucal, estratégias que busquem ampliar o acesso dos mais desfavorecidos são essenciais para a melhora dos indicadores de saúde bucal.
